# Supplementary material for: A printable hydrogel microarray for drug screening avoids false positives associated with promiscuous aggregating inhibitors
Source: Nat Commun. 2018 Feb 9;9:602. doi: 10.1038/s41467-018-02956-z (PMC5807445; doi:10.1038/s41467-018-02956-z)
Supplement: Supplementary file 1 — Supplementary Information [file 41467_2018_2956_MOESM1_ESM.pdf]

Supplementary Figures

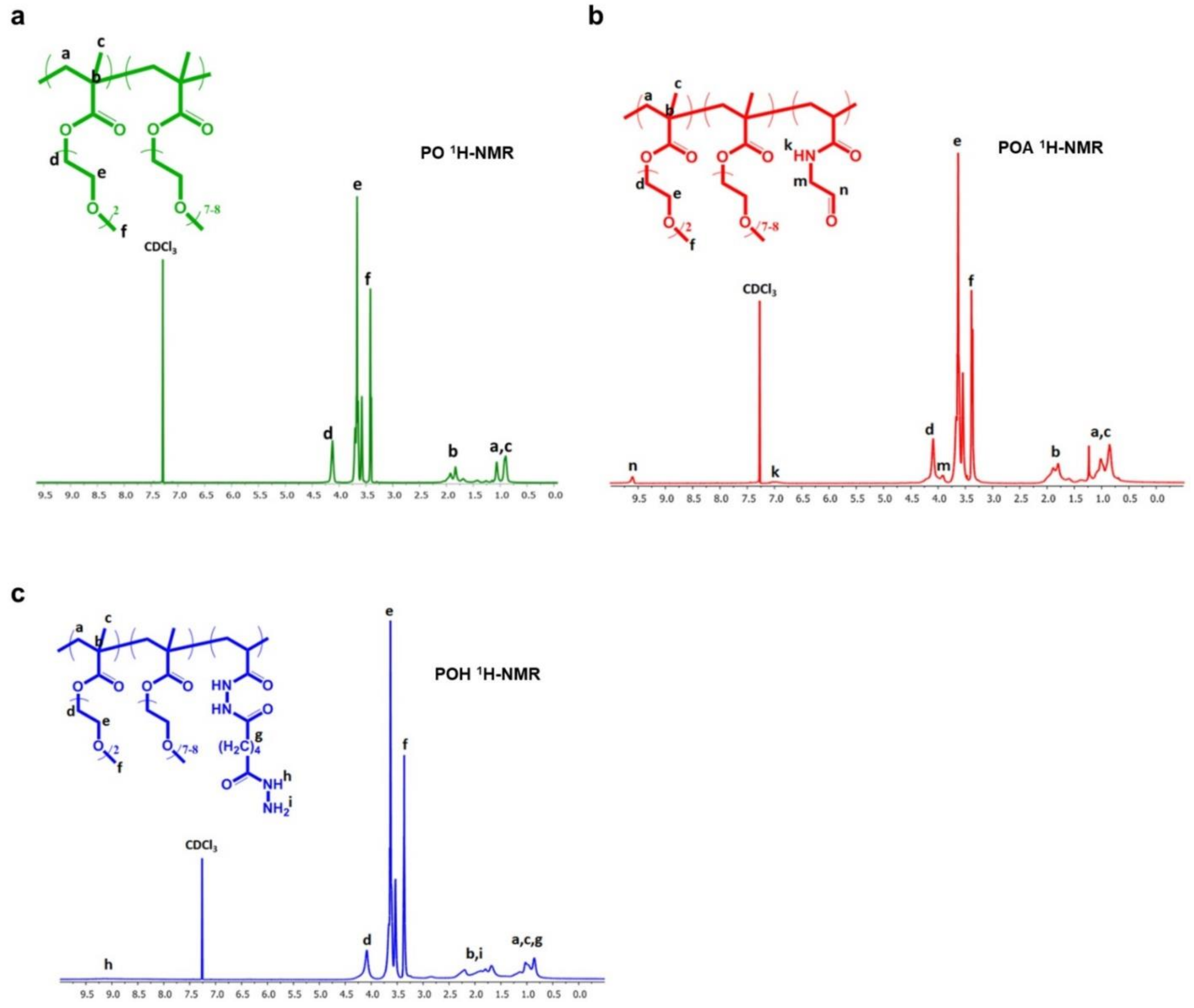

Supplementary Figure 1. ^{1}H-NMR spectra of polymers (a) Unfunctionalized poly(oligoethylene glycol methacrylate) (PO) (b) Aldehyde-functionalized poly(oligoethylene glycol methacrylate) (POA) (c) Hydrazide-functionalized poly(oligoethylene glycol methacrylate) (POH). Chemical shifts are reported relative to residual deuterated solvent peaks. Peak assignments are given on each spectrum based on the anticipated chemical structure of each polymer.

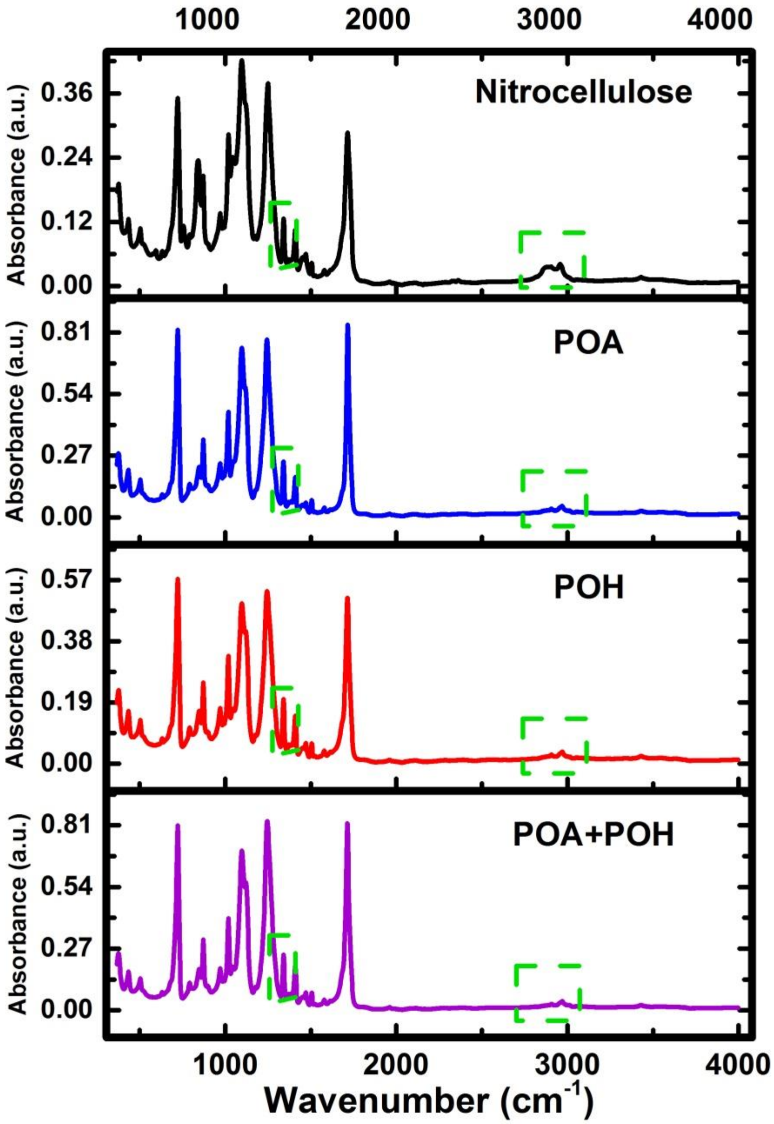

Supplementary Figure 2. ATR-FTIR absorbance spectra of printed polymers In the nitrocellulose spectrum, the peak at 1340 cm^{-1} corresponds to the nitro group (-NO_2) stretch while the peak at 2960 cm^{-1} corresponds to the -CH group stretch in the cellulose backbone (peak absorbance is measured in absorbance units (a.u.)). In the printed aldehyde-functionalized and hydrazide-functionalized poly(oligoethylene glycol methacrylate) (POA and POH, respectively) spectra, the peak at 1715 cm^{-1} corresponds to the ester group (-C=O) stretch from the poly(oligoethylene glycol methacrylate) (PO) polymer side chain. Hydrazide and aldehyde groups also both appear in the range of the ester signals and are convoluted with these ester peaks; however, the C=O signal is primarily associated with the PO polymers rather than the nitrocellulose. The decrease in intensity of both the nitro group and the cellulose -CH stretch peak relative to the carbonyl peak in the printed polymers suggests that the polymers were successfully printed onto the nitrocellulose paper surface.

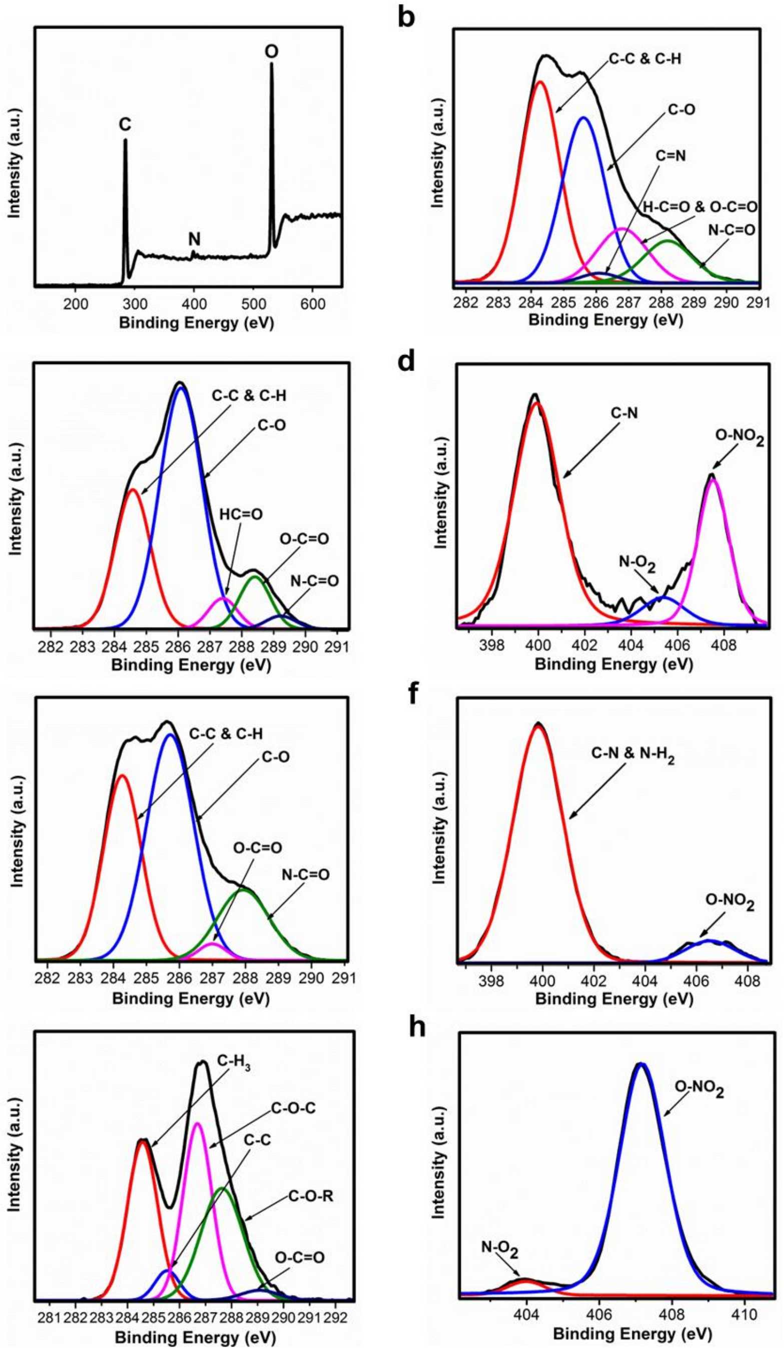

Supplementary Figure 3. High-resolution XPS spectra of printed polymers (a) Survey scan of POA+POH printed on a nitrocellulose substrate. (b) Spectrum of POA+POH printed hydrogel samples collected in the C 1s region. The peak at 286.1 eV corresponds to the –C=N group found in the hydrazone bond. (c) Spectrum of POA collected in the C 1s region. (d) Spectrum of POA collected in N 1s region. (e) Spectrum of POH collected in C 1s region. (f) Spectrum of POH collected in N 1s region. (g) Spectrum of nitrocellulose collected in C 1s region. (h) Spectrum of nitrocellulose collected in N 1s region.

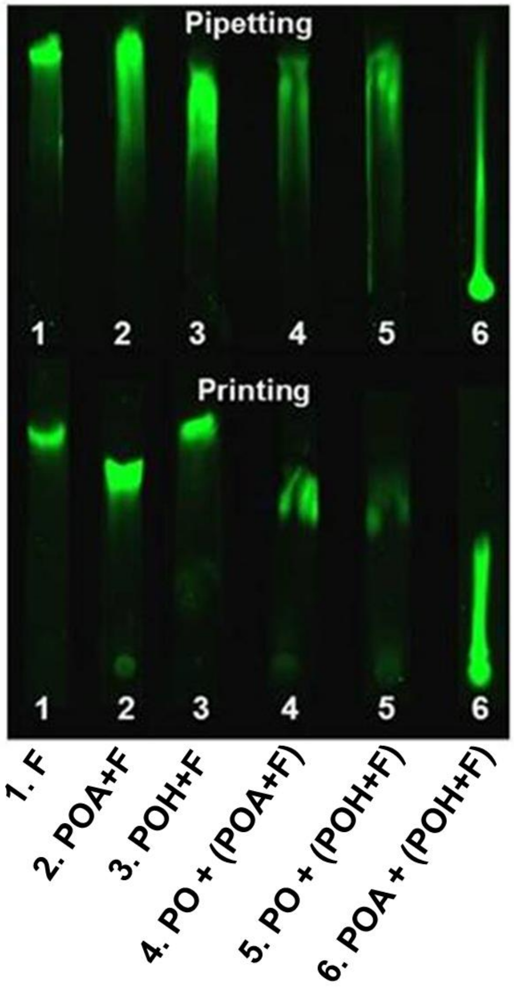

Supplementary Figure 4. Chromatography of polymers inks mixed with fluorescein (F) In the pipetting experiment, polymer inks were mixed with fluorescein and the corresponding reactive or unreactive polymer and directly pipetted onto a nitrocellulose paper substrate. In the printing experiment, reactive polymer inks (aldehyde-functionalized and hydrazide-functionalized poly(oligoethylene glycol methacrylate) (POA and POH, respectively) were mixed with fluorescein (F) and printed onto a nitrocellulose substrate, followed immediately by printing of the corresponding reactive polymer or an unreactive polymer (poly(oligoethylene glycol methacrylate) (PO). Chromatography was performed in a 50:50 methanol:water mixture. Lane 6 shows that the pipetted or printed polymer assembly (POA+POH) successfully immobilizes a large fraction of the fluorescein (with the portion eluting consistent with controlled release from a hydrogel), while all other samples tested result in transport of essentially all of the printed fluorescein up the strip, indicating a lack of effective encapsulation. Lane 1: fluorescein (F) only; Lane 2: aldehyde-functionalized poly(oligoethylene glycol methacrylate) mixed with fluorescein (POA+F); Lane 3: hydrazide-functionalized poly(oligoethylene glycol methacrylate) mixed with fluorescein (POH+F); Lane 4: (POA+F) printed on top of or pipetted with poly(oligoethylene glycol methacrylate) (PO+(POA+F)); Lane 5: (POH+F) printed on top of or pipetted with PO (PO+(POH+F)); Lane 6: (POH+F) printed on top of or pipetted with POA (POA+(POH+F)).

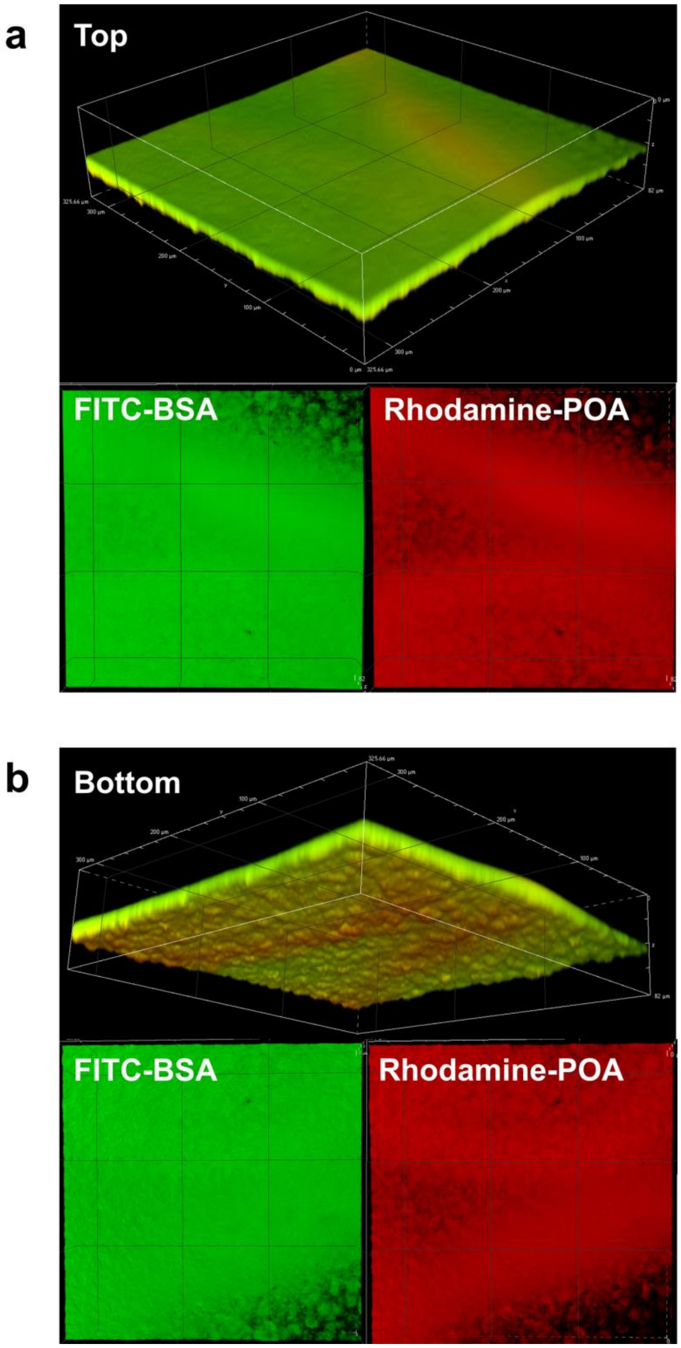

Supplementary Figure 5. Confocal microscopy of printed hydrogel microzones FITC (fluorescein isothiocyanate) channel (green), rhodamine channel (red), and overlaid fluorescence images confirm the co-localization of fluorescein isothiocyanate-labeled bovine serum albumin (FITC-BSA) within the gel phase as well as the relatively uniform distribution of FITC-BSA within the printed gel. The printed hydrogel was prepared by printing rhodamine-labeled aldehyde-functionalized poly(oligoethylene glycol methacrylate) (Rhodamine-POA) as the base polymer on a nitrocellulose substrate followed by hydrazide-functionalized poly(oligoethylene glycol methacrylate) (POH) ink (unlabeled) containing 0.005 mg mL^{-1} of FITC-BSA. Images represent the top (a) and bottom (80 μm depth) (b) of the 326 x 326 μm cross-sectional slice.

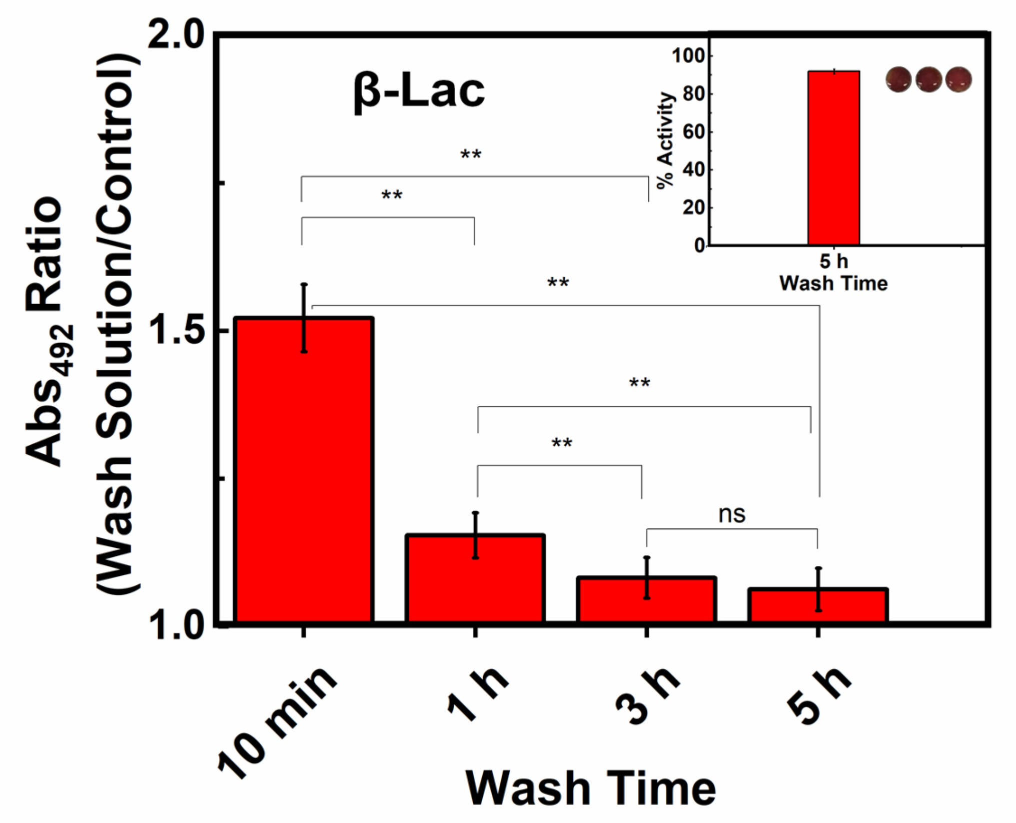

Supplementary Figure 6. Printing β-lactamase in hydrogel minimizes enzyme leaching
Printed samples were washed in 10 mM PBS for varying amounts of time. β-lactamase activity was then assessed in the wash solutions using UV-vis spectrophotometry to track the hydrolysis of nitrocefin by monitoring solution absorbance at 492 nm. The resulting absorbance readings are reported as a ratio of the control (i.e. the absorbance of buffer itself at 492 nm); as such an absorbance ratio of one indicates zero leached enzyme activity in the wash solution. Residual activity of samples washed for 5 h relative to the corresponding unwashed control is presented in the inset graph, confirming that minimal quantities of enzyme are leached from the printed hydrogel. Data are presented as means ± standard deviations (n=3); NS, not significant. ** $p<0.001$ by Student's *t*-test.

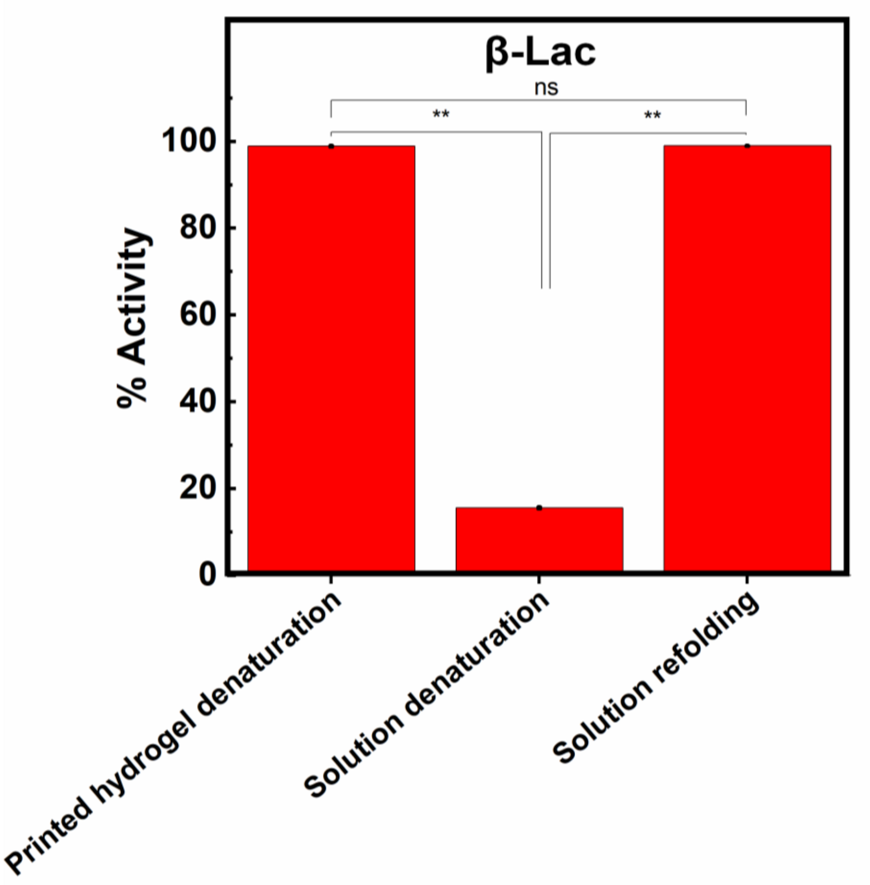

Supplementary Figure 7. Printed hydrogel protects β-lactamase against denaturation The remaining activity of β-lactamase printed in a hydrogel or in solution was quantified after 30 min. of treatment with urea denaturation buffer (a chaotropic agent, see Supplementary Methods) and normalized to the activity of the control incubated in 10 mM PBS. The solution refolding activity was measured by dialyzing a solution of β-lactamase prepared in urea denaturation buffer against 10 mM PBS in order to promote protein refolding and then re-testing the enzymatic activity; the full recovery of enzyme activity following dialysis confirms that urea was the driving force for the solution denaturation, as such denaturation is known to be reversible. Data are presented as means ± standard deviations (n=3); NS, not significant. ** $p<0.001$ by Student's *t*-test.

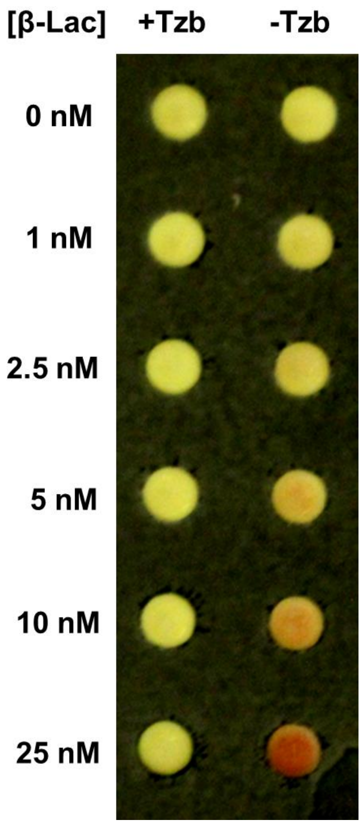

Supplementary Figure 8. Detection limit of β-lactamase drug screening assay A range of β-lactamase concentrations was encapsulated via printing in the POA+POH hydrogel, with the colorimetric readouts compared with and without tazobactam (a known inhibitor of β-lactamase, 100 µM concentration added). Significant and detectable colour differences were observed using enzyme concentrations as low as 5 nM, providing additional flexibility for the assay in detecting low K_i inhibitors.

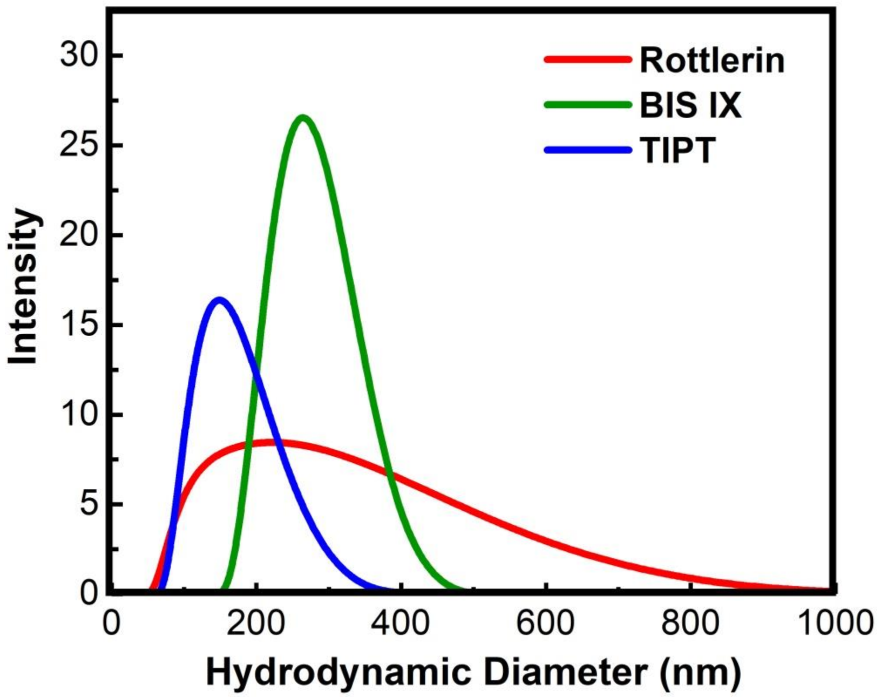

Supplementary Figure 9. Particle size distribution of aggregating inhibitors Intensity distributions of 100 μM solutions of rottlerin, bisindolylmaleimide IX (BIS IX) and tetraiodophenolphthalein (TIPT) were measured using dynamic light scattering (DLS). Each of these known promiscuous inhibitors shows clear evidence of aggregate formation on the order of 50-800 nm particle sizes, the full size range of which would be sterically excluded from diffusing into the printed hydrogel mesh.

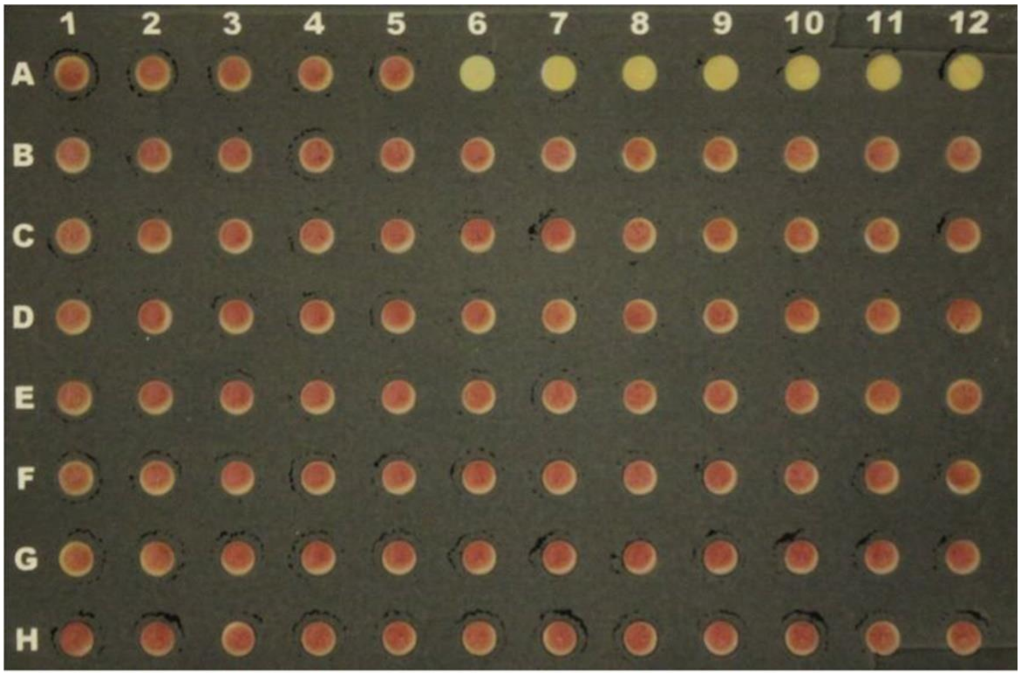

Supplementary Figure 10. A printable hydrogel microarray for drug screening Hydrogel spots entrapping β-lactamase were printed on microzones initially defined by wax-printing a 96-well pattern on nitrocellulose. The microplate has the same dimensions as a traditional 96-well microplate; smaller microplate mimics (e.g. 384 well formats) could similarly be printed if desired, as the resolution of the printing process is sufficient for such fabrication. The assay is performed by a liquid handling robot that dispenses inhibitor solutions and the enzymatic substrate onto the microzones.

Supplementary Tables

Supplementary Table 1. Enzyme concentrations in hydrazide-functionalized poly(oligoethylene glycol methacrylate (POH) inks

**Enzyme**	**Concentration in POH Ink ($U\ \mu L^{-1}$)**
Alkaline phosphatase (AP)	0.5
Urease	0.2
β-lactamase (β-Lac)	0.02

Supplementary Table 2. Substrates and added volumes used for each enzyme studied in this work

**Enzyme**	**Substrate**	**Volume of Substrate Added (μL)**
Alkaline phosphatase (AP)	BCIP®/NBT-Purple Liquid Substrate System	10
Urease	0.5 mM acetic acid, 5 mM urea, 0.005% phenol red	20
β-lactamase (β-Lac)	500 μM nitrocefin (19.4 μM DMSO stocks diluted in 10 mM PBS)	10

Supplementary Table 3. Size and polydispersity of aggregating inhibitors The aggregate size was measured in 100 μM solutions of rottlerin, bisindolylmaleimide IX (BIS IX) and tetraiodophenolphthalein (TIPT) using dynamic light scattering (DLS).

**Inhibitor**	**Size (nm)**	**Polydispersity**
Rottlerin	188 ± 1	0.24 ± 0.01
BIS IX	365 ± 9	0.36 ± 0.02
TIPT	154 ± 1	0.16 ± 0.02

Supplementary Methods

Materials

Oligo(ethylene glycol) methyl ether methacrylate (OEGMA, M_n = 475 g/mol, Sigma Aldrich, 95%) and (diethylene glycol) methyl ether methacrylate ($M(EO)_2MA$, Sigma Aldrich, 98%) were purified on a column of basic aluminum oxide (Sigma Aldrich, type CG-20) to remove the inhibitors methyl ether hydroquinone (MEHQ) and butylated hydroxytoluene (BHT) respectively. N-(2,2-dimethoxyethyl)methacrylamide (DMEMAm) was synthesized as previously reported (see N.M.B. Smeets et al., *Chem. Comm*., 2014, *50*, 3306–3309). Acrylic acid (AA, Sigma Aldrich, 99%), thioglycolic acid (TGA, Sigma Aldrich, 98%), 2,2-azobisisobutryic acid dimethyl ester (AIBMe, Wako Chemicals, 98.5%), adipic acid dihydrazide (ADH, Alfa Aesar, 98%), N'-ethyl-N-(3-dimethylaminopropyl)-carbodiimide (EDC, Carbosynth, Compton CA, commercial grade), sodium cyanoborohydride ($NaBH_3CN$, Sigma Aldrich, reagent grade), aminoacetaldehyde dimethyl acetal (Sigma Aldrich, 99%), 2,2,6,6-tetramethyl-1-piperidinyloxy (TEMPO, Sigma Aldrich, 98%), methacryloyl chloride (Sigma Aldrich, purum), fluorescein-5-isothiocyanate (5-FITC, Sigma Aldrich, 90%), rhodamine 123 (Sigma Aldrich, 85%) and bovine serum albumin (BSA, Sigma Aldrich, >96%) were all used as received. Hi-Flow plus cellulose ester membranes (EMD Millipore, HF12002XSS), glycerol (Sigma Aldrich, ≥99%), fluorescein (free acid) (Sigma Aldrich, 95%), alkaline phosphatase (AP, Roche, 20 U/μL), BCIP®/NBT-Purple Liquid Substrate System (Sigma Aldrich), urease from *Canavalia ensiformis*, Type III (Sigma Aldrich, 20 KU), phenol red solution (Sigma Aldrich, 0.5%), urea (Sigma Aldrich, ≥98%), recombinant β-lactamase TEM precursor from *Escherichia coli* (ProSpec, 700 U/mg), nitrocefin (Abcam, >95%), proteinase K (ThermoFisher, 20 mg/mL), tazobactam sodium salt (Abcam, >99%), sulbactam sodium salt (Santa Cruz Biotechnology), clavulanic acid potassium salt with cellulose, 1:1 w/w (Santa Cruz Biotechnology), rottlerin (Abcam, >95%), bisindolylmaleimide IX (BIS IX, Abcam, >98%) 3′,3″,5′,5″-tetraiodophenolphthalein (TIPT, Santa Cruz Biotechnology, >95%), 1,4-dithiothreitol (Sigma Aldrich, ≥97%), TRIS hydrochloride (Sigma Aldrich, >99%), sodium chloride (Sigma Aldrich, ≥99%) and Slide-A-Lyzer™ MINI Dialysis Devices (0.1 mL, 3.5 kDa MWCO, ThermoFisher) were all used as received. For all experiments, Milli-Q grade distilled deionized water (DIW) was used. Phosphate buffered saline (PBS) was diluted from a 10X liquid concentrate (Bioshop Canada Inc.).

Synthesis of poly(oligoethylene glycol methacrylate) polymers

Unfunctionalized poly(oligoethylene glycol methacrylate) (PO) was prepared by adding AIBMe (50 mg, 0.22 mmol), $OEGMA_{475}$ (0.90 g, 1.9 mmol), $M(EO)_2MA$ (3.1g, 16.5 mmol) and TGA (7.5 μL, 0.15 mmol) to a 50 mL Schlenk flask. 1,4-Dioxane (20 mL) was added, and the solution was purged with nitrogen for 30 minutes. The flask was sealed and submerged in a pre-heated oil bath at 75°C for 4 hours under magnetic stirring. After polymerization, the solvent was removed

by rotary evaporation, and the poly(OEGMA$_{475}$-co-M(EO)$_2$MA) polymer was purified by dialysis against DIW for 6 cycles (6 hours/cycle) and lyophilized to dryness. The polymer was dissolved in 10 mM PBS at 20 w/w% and stored at 4°C.

Aldehyde-functionalized poly(oligoethylene glycol methacrylate) (POA) was prepared similarly to the unfunctionalized PO polymer above except for the addition of DMEMAm (0.63 g, 3.61 mmol). Following solvent removal, the acetal groups of the DMEMAm residues were converted to aldehydes via hydrolysis by dissolving the copolymer in 100 mL of 0.25 M HCl and stirring for 24 hours. The polymer was purified by dialysis against DIW and lyophilized to dryness. POA was dissolved in 10 mM PBS at 20 w/w% and stored at 4°C. The number-average molecular weight was determined to be 14 kDa ($Đ$ = 2.03) from size exclusion chromatography. The aldehyde content was determined to be 12 mol% using ^{1}H-NMR by comparing the integration of the proton signals of the methoxy (O-CH_3, 3H, δ = 3.3 ppm) and aldehyde (CHO, 1H, δ = 9.2 ppm) groups.

Hydrazide-functionalized poly(oligoethylene glycol methacrylate) (POH) was prepared by adding AIBMe (37 mg, 0.16 mmol), OEGMA$_{475}$ (0.90 g, 1.9 mmol), M(EO)$_2$MA (3.1g, 16.5 mmol), AA (0.55 g, 7.6mmol), and TGA (7.5 µL, 0.15 mmol) to a 50 mL Schlenk flask. Polymerization proceeded similarly to that of PO and POA. Following solvent removal, the copolymer was dissolved in 100 mL DIW. ADH (4.33g, 24.8 mmol, 8.16 mol eq.) was added, and the pH of the solution was adjusted to 4.75. The reaction was initiated by the addition of EDC (1.93 g, 12.4 mmol, 3.80 mol eq.), after which the pH was maintained at 4.75 by the dropwise addition of 0.1 M HCl over 4 hours. The solution was left to stir overnight, dialyzed against DIW over 6 cycles (6 hours/cycle) and lyophilized to dryness. The polymer was dissolved in 10 mM PBS at 20 w/w% and stored at 4°C. The number-average molecular weight was determined to be 17 kDa ($Đ$ = 2.08) using size exclusion chromatography. The degree of hydrazide functionalization was determined to be 22 mol% by conductometric base-into-acid titration, comparing the carboxylic acid content before and after ADH conjugation (0.1 M NaOH titrant, 50 mg polymer in 50 mg of 1 mM NaCl titration solution, ManTech automatic titrator).

Polymer characterization

Size exclusion chromatography (SEC) was performed using a Waters 2695 separations module equipped with a Waters 2996 photodiode array detector, a Waters 2414 refractive index detector, a Waters 2475 multi λ fluorescence detector and four Polymer Labs PLgel individual pore size columns maintained at 40 °C, using a 5 µm bead size and pore sizes of 100, 500, 103 and 105 Å. THF was used as the eluent at a flow rate of 1.0 mL min^{-1}, and poly(methyl methacrylate) standards were used for calibration. ^{1}H-NMR was performed using a Bruker AVANCE 600 MHz spectrometer using deuterated chloroform as the solvent.

Fluorescent labelling of polymers and bovine serum albumin (BSA)

Fluorescein isothiocyanate (FITC) was conjugated to POH (FITC-POH) by reacting 1 g of POH was reacted with 5 mg of FITC in water at pH=8, stirring overnight at room temperature (2 mol% hydrazide groups labeled). Rhodamine 123 was conjugated to POA (Rhodamine-POA) via reductive amination, reacting 1 g of POA with 5 mg of Rhodamine 123 in water for 12 hours and then reducing the resulting Schiff base with sodium cyanoborohydride (8.25 mg, 10-fold molar excess to the rhodamine) to create a stable conjugate. Both labeled polymers were dialyzed against DIW for 6 cycles (6 hours/cycle) in the dark, lyophilized to dryness, dissolved in 10 mM PBS at 10% w/w, and stored at 4°C in the dark.

FITC-BSA was prepared by dissolving 50 mg of BSA and 1 mg of FITC in 100 mL of aqueous carbonate buffer at pH = 9.0. The reaction was carried out for 12 hours under magnetic stirring. The FITC-labelled protein was purified by dialyzing against DIW for 6 cycles (6 hours/cycle) and then lyophilized. FITC-BSA was stored at 4°C in the dark.

Characterization of printed hydrogels

ATR-FTIR was performed on printed polymer samples following extensive washing with 10 mM PBS using a Vertex 70 FTIR Diamond ATR (Bruker). XPS spectra were recorded with a Physical Electronics (PHI) Quantera II spectrometer using a monochromatic Al K-α X-ray source (1486.7 eV) at 50 W (15 kV). Survey (280 eV pass energy), high-resolution carbon (26 eV pass energy) and high-resolution nitrogen (55 eV pass energy) XPS scans were obtained using a 45° take-off angle. Data analysis was performed using PHI MultiPak software. Peak assignments were made according to the values reported in the NIST XPS Database. The surface morphology of both printed and non-printed surfaces was evaluated by scanning electron microscopy (SEM) (FEI-Magellan XHR) using secondary electron image (SEI) mode with voltages of 2.0 kV (1000x magnification).

Printed polymer chromatography

Rhodamine-POA or FITC-POH were printed alone, with PO (unfunctionalized poly(oligoethylene glycol methacrylate)) or with the corresponding, unlabelled reactive polymer. Paper samples were cut into 0.5x4.5 cm strips, and chromatography was subsequently performed by placing the end of each strip in 50 µL of a 70:30 methanol:water solvent mixture. The samples were imaged through the fluorescein and rhodamine channels of the ChemiDoc™ MP System (BioRad). Image processing was performed in Image Lab™ software (BioRad).

Protein adsorption

Printed POA/POH hydrogels were soaked in 10 mM PBS for 12 hours, after which the hydrated samples were submerged in a 100 μg mL^{-1} solution of FITC-BSA and gently shaken for 2 hours. The samples were imaged through the fluorescein channel of the ChemiDoc™ MP System (BioRad). Image processing was performed using Image Lab™ software (BioRad).

Fluorescein and FITC-BSA entrapment

POH ink solutions were co-dissolved with 10 μM fluorescein or 0.05 mg mL^{-1} FITC-BSA. Samples printed with fluorescein were washed in 0.1 M NaOH + 0.1% Tween 20, while samples printed with FITC-BSA were washed in 10 mM PBS and shaken at 300 rpm on an IKA MS3 Basic Shaker for 30 minutes.; each rinse solution was selected to maximize the solubility of the fluorescently-labeled probe and thus maximize the potential for washing the probe away from the surface if it was not effectively immobilized. Afterwards, both samples were imaged through the fluorescein channel of the ChemiDoc™ MP System (BioRad). Image processing was performed in Image Lab™ software (BioRad). FITC-BSA printed samples were also imaged with a Nikon Eclipse LV100ND optical microscope equipped with an Andor Zyla sCMOS camera at 20x magnification through the fluorescein channel to assess the distribution of FITC-BSA on the printed hydrogel surface.

Chromatographic experiments confirming immobilization of encapsulated fluorophores upon gel printing were additionally performed by printing the relevant POA or POH solutions on a nitrocellulose paper substrate as described above, cutting the printed paper into 0.5x4.5 cm strips, and performing chromatography by dipping the end of the strip in 50 μL of a 50:50 methanol:water solvent. The samples were imaged through the fluorescein channel of the ChemiDoc™ MP System (BioRad). Image processing was performed using Image Lab™ software (BioRad).

The distribution of Rhodamine-POA and FITC-BSA within the printed gel layer was assessed using confocal fluorescence microscopy (CLSM, Nikon). Confocal z-stack images (3D view) were collected by scanning the printed gel samples at 2 µm intervals to a depth of 80 μm (326 x 326 µm area probed). Excitation/emission wavelengths of 488/525 nm (FITC-BSA) and 561/595nm (Rhodamine-POA) were used to acquire the images.

Enzyme leaching study

1 μM β-lactamase was printed in the PO-based hydrogel, followed by washing of the printed samples in 10 mM PBS for varying amounts of time. β-lactamase activity was assessed in the wash solutions by adding nitrocefin to a final concentration of 200 μM and tracking the hydrolysis of nitrocefin by monitoring solution absorbance at 492 nm via UV-vis spectrophotometry (Infinite M1000 spectrophotometer, Tecan). The resulting absorbance readings were reported as a ratio relative to the control (the absorbance of buffer itself at 492 nm, without any substrate added). Subsequently, the residual activity of the printed samples washed over 5 h was measured via image acquisition and analysis as described for the entrapment studies, with the results presented as a ratio of the corresponding control image (a printed hydrogel sample not washed to remove any non-adsorbed enzyme).

Denaturation study

For the printed hydrogel denaturation study, 10 μL of urea denaturation buffer (8 M urea, 5 mM dithiothreitol, 50 mM Tris-Cl (pH=7.5), 150 mM NaCl) was pipetted onto samples of 1 μM β-lactamase entrapped in the printed hydrogel. The samples were incubated in a closed container for 30 min. at room temperature and then washed with DIW. Image acquisition and analysis was performed as described for the entrapment studies. The intensity of each sample was measured and presented as a ratio of the corresponding control image (samples treated with 10 mM PBS). For the solution denaturation study, 1 μM β-lactamase was prepared in 100 μL of urea denaturation buffer and incubated for 30 min. at room temperature, after which nitrocefin was added to a final concentration of 200 μM. β-lactamase activity was then assessed via UV-vis spectrophotometry, tracking the hydrolysis of nitrocefin (Infinite M1000 spectrophotometer, Tecan) by monitoring solution absorbance at 492 nm. For the solution refolding study, 1 μM β-lactamase samples prepared in urea denaturation buffer were dialyzed against 10 mM PBS using a 3.5 kDa MWCO dialysis device (ThermoFisher) for 20 cycles (20 min/cycle). β-lactamase activity was then re-assessed via UV-vis spectrophotometry as described above.

Optimizing the β-lactamase concentration in a printed hydrogel-based screening assay

β-lactamase concentrations ranging from 0 to 25 nM were printed in the PO-based hydrogel as described previously. Each microzone was treated with 100 μM tazobactam (5 μL) or water (5 μL) for 20 min., followed by the addition of 500 μM nitrocefin (5 μL). Images of the microzones were taken with a Canon DSLR camera (operated in manual focus mode without flash) 25 min. after substrate addition and analyzed via image analysis as previously described.

Promiscuous aggregating inhibitor size measurement

Dynamic light scattering measurements were performed using a Zetasizer Nano ZS instrument (Malvern). The intensity of backscattered light was measured at a 173° angle. Data collection and analysis were performed using the Dispersion Technology Software (version 6.0) from Malvern. Samples of Rottlerin, BIS IX and TIPT at 100 μM concentrations were analyzed in a plastic cuvette with a 10 mm path length. Prior to measurement, samples were equilibrated for 120 s at 23°C. For each sample, 10 runs were performed with three repetitions. The intensity size distribution, Z-average diameter and polydispersity index was collected for each sample. Note that no significant signal was acquired from DLS measurements on the true inhibitors, confirming their solution state.
